# Collaboration with people with lived experience of prison: reflections on researching cancer care in custodial settings

**DOI:** 10.1186/s40900-021-00284-z

**Published:** 2021-06-29

**Authors:** Renske Visser, Alyce-Ellen Barber, Anthony X, Sue Wheatcroft, Philip Mullen, Jo Armes

**Affiliations:** 1 University of Surrey, School of Health Sciences, Guildford, UK; 2 Revolving Doors Agency, 90 London Rd, Elephant and Castle, London, SE1 6LN UK

**Keywords:** Peer research, Co-production, Cancer, Prison, Lived experience, Experts by experience, Healthcare, Patient and public involvement

## Abstract

**Background:**

Patient and public involvement is increasingly considered important in health research. This paper reflects, from both academic and lived experience perspectives, on involving people with lived experience in a study exploring cancer care in prison and how by doing this it enriched the research process.

**Methods:**

This paper is based on written and verbal reflections of the lived experience researchers and academic researchers involved in a study exploring the diagnosis and treatment of people with cancer in prison. The study comprised interviews with people with cancer in prison, prison healthcare staff, oncology specialists and custodial staff. Lived experience researchers were involved throughout the research process, including co-conducting interviews with patients and analysing interviews.

**Results:**

This paper highlights the importance and value of including lived experience researchers across the research process. We reflect on how lived experience of prison shapes the experience of conducting interviews and analysing data gathered in prison. We reflect on the working relationships between academic and lived experience researchers. We demonstrate how prison research is challenging, but collaboration between lived experience and academic researchers can help to better prepare for the field, to ask more meaningful questions and to create rapport with participants. These types of collaborations can be powerful avenues for skill development for both academic and lived experience researchers, but they require an investment of time and a willingness for shared learning.

**Conclusions:**

For academics and lived experience researchers to collaborate successfully and meaningfully care needs to be taken to develop open, honest and equal working relationships. Skills development for academic and lived experience researchers is important. A commitment to building and maintaining relationships is crucial. Having a third party as a mediator can facilitate and foster these relationships. Particularly with people with lived experience of prison it is essential to put the ‘do no harm’ principle into practice and to have support in place to minimise this.

**Supplementary Information:**

The online version contains supplementary material available at 10.1186/s40900-021-00284-z.

## Plain English summary

While patient and public involvement is increasingly considered important in health research, very few papers reflect on the process of collaborating with people with lived experience of prison in health research. This paper is based on written and verbal reflections of both the lived experience and academic researchers on the project that explored how cancer is diagnosed and treated in prison. For academics and lived experience researchers to collaborate successfully and meaningfully care needs to be taken to develop open, honest and equal working relationships. Skills development for academic and lived experience researchers is important. A commitment to building and maintaining relationships is crucial. Having a third party as a mediator can facilitate and foster these relationships. Particularly with people with lived experience of prison it is essential to put the ‘do no harm’ principle into practice and to have support in place to minimise this. The process of writing this paper provided additional opportunities to reflect on the collaboration which we all found vital. Involving lived experience researchers on all aspects of the research process can strengthen the design, relevancy and outcomes of studies. The process, however, can be emotionally challenging for lived experience and academic researchers, underscoring the need for space for open and honest reflection and learning. Particularly for people with lived experience of prison, being involved in research studies can be a great source of personal growth as it offers an opportunity to use and reframe their, often traumatic, lived experience in a positive way.

**Supplementary Information:**

The online version contains supplementary material available at 10.1186/s40900-021-00284-z.

## Introduction

While the involvement of lived experience researchers in health research is increasingly common [1, 2], the involvement in health research of people who also have lived experience of the criminal justice system is still rare [3, 4]. Research that involved people with lived experience of secure environments as peer researchers has highlighted the difficulty of getting access to this group, and the complexity of establishing and sustaining relationships [3, 4]. Whilst involving patients and the public in research can be beneficial, they can also feel used [5]. This is particularly the case in prison settings where involvement has been identified as being akin to tokenism [6]. Buck et al. [6] therefore outline three specific principles when involving people with lived experience of prison in research: 1) to avoid tokenism and to be sensitive to the needs of people sharing their shame or trauma experiences, 2) to learn to leverage the engagement of marginalised people in research in movements for change, and 3) to acknowledge there is no singular lived experience and therefore include a range of voices in research. As Earle [7] points out “*Ex-prisoners are not a homogeneous group and prison experiences vary according to one’s gender, age, ethnicity, and class. They also vary by sentence length, offense category, and the kind of prisons served in*” (p.434).

An INVOLVE guidance report (2018, 4) [8] outlines the following key principles for good collaborative research practice:

1. **Sharing of power** – the research is jointly owned and people work together to achieve a joint understanding.
2. **Including all perspectives and skills** – ensure the research team includes all who can contribute.
3. **Respecting and valuing the knowledge of all those working together on the research** – everyone is of equal importance.
4. **Reciprocity** – everybody benefits from working together.
5. **Building and maintaining relationships** – an emphasis on relationships is key to sharing power, including joint understanding and consensus over roles and responsibilities.

These principles are important in all collaborative research but take a different shape when involving peer researchers with lived experience of prison, as there are levels of stigma and/or trauma that may not be experienced by other groups of lived experience researchers. At the same time involvement in research can be a means for lived experience researchers to feel empowered and to learn new skills [1]. Nevertheless collaboration can be ‘messy’ [9].

In our study investigating cancer care in prison, academic researchers (JA and RV) collaborated with three Experts by Experience (AX, AB, SW), or lived experience researchers, from Revolving Doors Agency (RDA). Revolving Doors Agency (RDA) is a UK national charity that seeks to create a smarter criminal justice system that makes the revolving door of crisis and crime avoidable and escapable.^1^ RDA combine lived experience insight, robust research and system knowledge to co-create policy and practice solutions that work. This paper aims to encourage others to harness the benefits of co-producing research with people with lived experience of health care in prison, in a safe and genuine way, by offering our reflections on the process.

Whilst we do not report findings from the larger study in this paper, the aim of the study was to explore how people in prison are diagnosed with and treated for cancer to better understand how these processes work and can be improved.^2^ The qualitative phase comprised interviews with people with cancer in prison, prison healthcare professionals, custodial staff and oncology clinicians. From the start of the project, Experts by Experience were involved as advisors on the project. They helped design information sheets and interview schedules for the patient group and answered many practical questions about being in a prison environment that helped us to think about how we could conduct interviews sensitively. Additionally, they participated in project team meetings to ensure the voices of people in prisons were not forgotten. Due to the good working relationship between researchers at the University of Surrey and Experts by Experience from RDA it was agreed that Experts by Experience would be involved in a more collaborative role in later stages of the research, including patient interviews and data analysis.

We agree with Pearce [10], that research with lived experience researchers is strongest when it is *relational:*

*“ PPI/co-production activities should be measured and judged in accordance with the ways in which relationships develop and change, and how those relationships inform how decisions, ideas and research knowledge are constructed.”*

This paper reflects on the collaboration between lived experience researchers and academic researchers. Topics that we discuss include: the emotional labour of prison research; mixed emotions about returning to ‘your’ prison; co-conducting interviews in prison; how lived experience can shape different interpretations of interview data compared to academic researchers; how involvement in research can be both challenging and meaningful for lived experience researchers. We conclude by offering lessons learned from the collaboration between academic and peer researchers, for example the importance of investing time and energy in establishing good working relationships between academic and lived experience researchers.

## Methods

This paper is based on the reflections of the Experts by Experience, hereafter referred to as “lived experience researchers”, and explores their involvement and personal growth throughout the study. These reflections were generated in various ways: lived experience researchers were encouraged to keep a research diary; post-interview debriefing sessions; four group discussion sessions between academic and lived experience researchers; and individual written reflections on topics discussed as a group. We will present excerpts from written and verbal accounts to illustrate the topics discussed. To offer a balanced account, reflections of both the lived experience researchers and academic researcher (RV) are used to highlight the strengths and learning points of what it means in practice to include lived experience researchers in prison healthcare research. The process of writing this paper was an important part of the reflexive process and formed part of the analysis. Researcher RV collated the individual reflections and drafted the initial paper. All the other authors then contributed and commented on this draft and the themes presented below were agreed upon by the entire research team.

### Process and outcomes

#### Background of the lived experience researchers

The three lived experience researchers all brought their own experiences to the project. Two were women and one was a man.^3^ During an initial training session we explored why they had chosen to support the project and how they felt their perspective might affect how they approached interviewing and analysis. For example, SW made the following comment:

“*I had known two people in prison with cancer. Both of them were released to a hospice shortly before they died, although one, Kay,*^4^*had deliberately offended in order to be sent back to prison, to spend her last few weeks surrounded by her friends. Her death had an enormous impact on me, not least because I saw how much pain she was in, with only a bottle of Oramorph for relief because intravenous pain relief was not allowed. I saw my part in this project as both helping to ascertain what was right/wrong with cancer care in prison and being part of a something that could bring about positive change, where necessary*”

SW’s personal experience of healthcare, and that of her friends, in prison was predominantly negative, and the limited access to pain relief in prison awakened a need for activism. This meant SW approached interviews in which patients in prison recounted quite positive care experiences with a level of scepticism and cynicism. AB, similarly, had a negative experience with prison healthcare:

“*When I was in prison I was diagnosed with auto immune diseases which need to be controlled with immunosuppressants, mainly methotrexate, which is a DMARD or a very low dose chemotherapy drug. I was told when I was inside, that I wouldn’t be able to take this while I was there, because they didn’t want me to be “sick on their time*”. (AB)

SW comments on her perception that prison staff respond to physical and mental health complaints differently:

“*My personal experiences of prison healthcare had been rather negative because of the stigma surrounding mental health, which is perhaps why one thing that stood out to me was the ‘acceptance’ by others, that these patients experienced in having an indisputable illness such as cancer. My experience of observing women in prison who had regular episodes of epilepsy, was that they were regularly treated as attention-seekers and left to deal with it themselves*.” (SW)

These first-hand experiences shaped their knowledge of healthcare in prison and influenced how they approached the research topic. It also formed the basis of research team discussions and points to the importance of a shared space for reflection between academic researchers and lived experience researchers. At times, lived experience researchers were very sceptical of information presented, whilst perhaps the academic researchers were not sceptical enough (we reflect on this further below). It was only through discussion that these diverging perspectives came to the fore, demonstrating the importance of having these types of discussions regularly.

#### Preparing for the field

Providing lived experience researchers with the qualitative research skills needed for the research is important [4]. Previous research has shown that inadequate training meant that peer researchers felt they could not contribute [1]. To prepare the lived experience researchers for the interviews with cancer patients in prison, a research training day was organised. The concept of reflexivity was introduced as follows:

*Reflexivity can be defined as thoughtful, conscious self-awareness. Reflexive analysis in research encompasses continual evaluation of subjective responses, intersubjective dynamics, and the research process itself. It involves a shift in our understanding of data collection from something objective that is accomplished through detached scrutiny of “what I know and how I know it” to recognizing how we actively construct our knowledge* ([11], 532).

Lived experience researchers were encouraged to reflect on their personal experiences and how these might impact the research. The aim of the exercise on reflexivity was to highlight that lived experience researchers are more than ‘ex-offenders’, a term that can come with a lot of stigma. As AB notes: “*There will always be an air of judgement surrounding people in the criminal justice system and this judgement can affect how the results of work are presented as well as received*.” As this project explored the relationship between healthcare and prisons, RDA selected lived experience researchers according to their experience of healthcare problems in prison and/or experience of assisting others with health problems in this environment.

Training in ‘what a semi-structured interview is’ and ‘how to ask open-ended questions’ was provided and the day ended with a roleplaying exercise in which each lived experience researcher practiced their interview skills. As the background of lived experience researchers is diverse, they can have wide ranging research experiences. Exploring the previous research experience of lived experience researchers is therefore important. This helps academic researchers to prepare lived experience researchers for the role they will play in individual research projects, and to tailor information and training to their specific skillsets and needs. Communication with lived experience researchers is key otherwise the training could come across as patronising or be overwhelming, if not at the right level for particular lived experience researchers.

#### Returning to prison

The lived experience researchers were involved in conducting the interviews with cancer patients in prison. This meant returning to prison, which was not always easy for them. It can evoke bad memories and mixed emotions. AX was particularly worried that he would not be allowed back into a prison because of his history and so got a new passport specifically for this project, as he knew he would not be allowed in without valid ID. On the day of the interviews he brought both his passport and birth certificate as he was convinced he needed as much proof as possible. Otherwise he feared he would be refused entry at the gate:

“*I was thinking that RV was naïve, that I would be let into the prison in the first place, I thought they would be looking for an excuse to knock me back – I thought the new passport might be used as an excuse to not let me in*” (AX)

Worry about being refused entry dominated his thoughts, leaving no room to worry about anything else. He was surprised how smooth his entry into the prison was in practice, and that no one challenged his presence.

Researcher RV, on the other hand, worried about AX once they were inside. While she wore her University photo ID, they were not given visitor badges or something to signify that they were not from the prison. As they entered a male prison, and one AX had spent time in, she worried prison officers might recognise him and not let him out again. Furthermore, because AX had been in this prison, another worry was he might panic when they were inside and/or want to leave. RV reiterated AX should let her know if he wanted to leave but as this was her first time inside a prison she did not know how easily they could have turned around and left.^5^

*“There is additional emotional labour when working with lived experience researchers. On interview days I was not only worried for my own feelings but also worried about the responses of lived experience researchers to the environment. Looking back, I feel it is a weird mix of feeling responsible for someone else, and feeling you, for some reason, should be able to cope with things better because you are an “academic”. As prison is such a strange research environment, I was relieved that I did not conduct the interviews on my own and shared this experience with lived experience researchers” (RV).*

As this research study focused on the healthcare experiences of people in prison, the researchers were not interested in the reason why participants were in prison. This was a deliberate research strategy. They never asked participants why they were in prison. In one interview conducted by AX and RV the interviewee started to talk about his reason for being in prison. Academic researcher RV comments:

“*I was impressed by the way AX deflected that situation. AX was leading the questions and I remember becoming tense when the interviewee started to describe why he was in prison, and wondered whether I should intervene. Very skilfully, and respectfully, AX stopped the interviewee and said “we are not here for that”, and continued the conversation.”*

Both AX and SW also returned to the prisons they had spent time in. AX notes:

“*On the way to the prison to meet the other researcher [RV], I felt mixed emotions; excitement, apprehension and fear. I walked alongside the huge enclosing prison wall when I had to take a moment and gather my thoughts. I imagined the faces of the front desk security looking at me and turning me away instantly, ‘you’re not welcome here’. Once inside I felt a surge of overwhelming joy. I have spent many wasted years in this place, and here I am now, a free man – a changed man. It felt surreal, especially with the responsibility of interviewing prisoners*.” (AX)

SW on returning to a women’s prison where she had spent a year of her life noted:

“*On the drive there that morning, I felt wary, but also, a little excited. I wondered if I would see anyone I knew. Rather disappointingly, I didn’t see anyone I knew. In fact, we didn’t see many prisoners or staff members. It was lock-up time and the only people on the wings were the cleaners and the occasional officer. Since that day, I have questioned why I was so disappointed that I hadn’t seen the people (officers and management) who had caused me so much misery.*”

The interview took place in a room very familiar to SW and she reflected on how this made her feel:

“*The interview with the female prisoner went well. It took place in the office of the manager with whom I had interacted many times, both in segregation and on the wings. When I entered the office, I wished he had been sitting there, behind his desk. I wanted the opportunity to shout at him, just as he had at me many times, to say ‘see, you were wrong about me!’* ”

Unfortunately, due to COVID-19, AB was not able to conduct interviews for this project. Reflecting on other projects she comments:

“*I have been in quite a few prison’s since leaving prison myself. It’s always been a weird experience, especially my first time back in a women’s prison. I found myself reverse quite easily back into the role as a prisoner. Whereas in the men’s prisons, I obviously was there for work, and not as a prisoner. I found being in a men’s prison slightly intimidating at first, but now it doesn’t faze me as much*.” (AB)

The experiences of the lived experience researchers show that participation in research needs careful consideration and preparation. Lived experience researchers can have a range of worries returning to prison. Guidance and support are needed, as lived experience researchers can be confronted with new scenarios or uncomfortable situations. As demonstrated by SW and AX, returning to prison as a researcher can equally have a beneficial impact, as it showed how their lives had changed for the better. This benefit is particularly pertinent for prison lived experience researchers. Having a history of being in prison can come with ongoing stigma or judgment from others and using new skills and contributing to meaningful research in an environment in which someone ‘wasted’ time can be especially cathartic.

#### The strength of co-conducting interviews with lived experience researchers

While lived experience researchers may not have the exact same lived experience as participants in the research, their presence in interviews can serve as a great equalizer. This is especially relevant for prison research as they are notorious for being places in which it is difficult to know who to trust. Disclosing their prison history was a way to instantly build rapport in our study. AX suggests it was a real benefit that he was one of the researchers, as he was familiar with prison jargon and he gave the participants a sense of safety:

“*Never mind the accents,*^6^*maybe the interviewees were feeling more confident speaking to me - for someone to relate to - you (RV) might have missed some things if I wasn’t there*”

During the interviews, AX took the lead in asking the questions. RV sometimes asked follow-up questions or checked the meaning of certain terms or acronyms. Even when she did ask the questions interviewees looked at AX when answering them. This might be to do with gender dynamics in the room, RV being a woman and AX a man, but may also indicate a level of trust between the participants and AX.

AX worried that he might encounter a participant he knew and often mentioned ‘he knew a guy with cancer in prison’. Little did he know that we would be sat face to face with this person on his first research day in prison.

“*One of my fears was knowing one of them, I was thinking they might have been like ‘Nah, you can’t be interviewing me, how’s this happening?! This is weird’. Thought they would be asking questions: who are you working with? How’s this happening? I can’t take this seriously – it could also have been someone I could have had a fight with. The person I knew gave the best interview in the end, even though he was difficult to understand*” (AX)

The worries AX expressed related to not feeling legitimate to conduct this research, yet the fact that he knew the person, provided a level of trust that other researchers could not have achieved. The prison nurse helping with the research was worried that this person might refuse to take part. Knowing AX was a great starting point for a very meaningful discussion.

When conducting interviews in prison, researchers have to work around prison regimes. Interview patterns, which are common when conducting interviews in the community, are difficult to replicate. For example, typically some time is spent getting to know each other, before doing the interview, and debrief with the interviewee afterwards. SW comments:

“*As a researcher going into prison, I realised that much depended on the availability of staff and that no-one, including visitors, could rely on things running smoothly. We soon came to realise that the interviewee could be taken back to his cell at any time; a time that was convenient for the staff rather than for us to finish the interview. I wasn’t surprised by this, but I did feel for the interviewees. Unfortunately, we were unable to make time for ‘niceties’, such as pre and post interview chats. I felt that was something we owed them. They were not paid anything for speaking to us and what they revealed, in relation to their healthcare as cancer patients, was likely to have been somewhat unsettling*” (SW)

In this study interviews were cut short on multiple occasions as participants had to return to their cell. Building trust quickly is thus essential and something lived experience researchers played a key role in.

#### New “lived experience”

Collaborating on prison research results in new “lived experiences” for lived experience researchers. As noted earlier, returning to prison can be very emotive, but it can also add a new layer to their understanding of the prison system. For SW taking part in the interviews revealed that the lack of communication and rigidness of the prison regime she experienced as a prisoner was similar for ‘outsiders’ like academic researchers:

“*When we arrived at the male prison, we found that we were not on the list of visitors. It was sorted out before long and I didn’t think too much about it. When we returned the next day, the same thing happened. I didn’t question it too much; I was preoccupied with the fact that I was being treated the same as the researcher I was accompanying. The staff were not particularly efficient, something I had got used to as a prisoner, but they were quite friendly, something I was not used to*.”

While the researcher got increasingly annoyed with ‘*not being on the list’*, SW reflects that this was “*still the nicest anyone has ever treated me in prison*”. Returning to prison in a researcher capacity reveals the power of labels such as ‘prisoner’ or ‘researcher’ and the different attitudes people have towards those groups. Similarly, AB comments:

“*I've definitely learned a lot about how security affects the treatment of prisoners. There is a part of me that feels like these things could potentially work differently. Also, because we as prisoners often aren’t aware of the security aspects of things, we feel angry at what seems like mistreatment, but really it's out of the hands of some officers and other staff.*”

AX compares his experience of being a researcher in prison with the feeling of being a prisoner taking part in research:

“*The feelings I went through wouldn’t have been anywhere near the same if I was a prisoner taking part in the research. It was very emotional leaving the jail after interviewing the prisoners. You left feeling drained. Whereas if I was a prisoner taking part in research all that emotion and exhaustion wouldn’t have been anywhere near what it was*”

SW also notes:

“*Going into the male prison was interesting and tiring but left no significant lasting impressions. Going into the female prison that I had spent time in as a prisoner, however, did have an impact on me and I think, probably, in a good way. I was able to exorcise some demons surrounding the prison officers I had known previously.*”

After each interview day a debrief was held in a café near the prison, and RDA conducted debriefing sessions over the phone. A year has elapsed since the first interview, but when discussing the interviews it can feel like they happened yesterday. It is therefore advisable to have ‘check-ins’ with lived experience researchers on a regular basis, even after the fieldwork part of research has finished.

### Lived experience involvement in research analysis

To create suitable interventions and meaningful change lived experience researchers should be involved in all stages of research, including analysis [12].^7^ As a research team we organised online analysis sessions.^8^ Interview transcripts were circulated prior to these meetings and coded individually by each research team member. Reading transcripts can be a powerful experience and AX notes:

“*Reading the transcript of the interview I had done was like I was back there, I could put myself back there. I felt a bit sad, could see the food he was referencing – which sends shivers down your back*”

For AX, the interview transcripts of ‘his’ interviews were more emotive than ones he had not conducted himself. He felt a distance towards the other interviews. But interview transcripts can evoke strong emotions, even when not having conducted the interviews yourself. AB commented:

“*While I expected to be emotionally affected by reading through the transcripts and the plights of others, I never thought I would be physically affected. I have numerous autoimmune diseases and to help control them, I inject a small dose of Methotrexate, a chemotherapy drug. Whilst reading one transcript, I found myself physically triggered and the side effects of my injection hit me quite hard and I had to stop and lay down”*

AB’s physical reaction on reading this interview demonstrates the ongoing embodied experience of being in prison and highlights that appropriate support needs to be in place so lived experience researchers can discuss these responses and feelings.

Analysing the patients’ interviews was easiest for the lived experience researchers, perhaps because these came closest to their own experiences:

“*Nothing came as much of a surprise to me. During my time in prison, I had met many women, experiencing a range of illnesses and conditions. Although most received adequate medical treatment, there was a general feeling of resignation when it came to the poor ‘bedside manner’ exercised by the prison staff. The following quote certainly didn’t surprise me… ‘I was fine throughout the treatment because I had no choice.’ As a former prisoner, I was angry at this. As a researcher, I was sad that it was still the same, and frustrated at the lack of progress.” (SW)*

#### Prison officer interviews

Prisons are places with complicated power dynamics, and the ‘us’ vs. ‘them’ culture between prisoners and prison officers is well documented [13]. This was something that was also felt by our lived experience researchers who discussed how they had difficulty in believing the accounts of the officers:

“*I think maybe this is why I struggle to take the transcripts of the officers as genuine. It all seems so scripted and the “decent thing to do*^9^*”. Can you be compassionate when your job, all your training and adult conditioning is to be cynical and disbelieve anything anyone has to say to you? I imagine prison officers wouldn’t even believe a prisoner had cancer if they told them themselves, not until they saw medical evidence.*” (AB)

“*Reading the transcripts made me look at myself, and any possible bias I may have held. Unlike the patient interviews, where I could give the benefit of doubt when being unsure, I struggled with this when it came to prison staff, whether in health or justice. This was most evident when reading the transcripts of interviews with the two prison officers. Both were confident that they understood the prisoners and did everything they could to help them, going the extra mile when others couldn’t or wouldn’t. I was doubtful about this and was obviously unsure how to interpret what I was reading, perhaps proving that one person’s lived experience and views alone, are not enough to gauge a true understanding of the transcripts*” (SW)

Being part of this research project complicated these feelings for the lived experience researchers as understanding the perspective of prison officers felt uncomfortable.

“*This may sound a strange thing to mention, but I had spent so many months with the attitude of ‘them and us’, I surprised myself and, to be honest, I felt conflicted. I felt a little guilty and disloyal to the women I had left behind and were still somewhere in the prison estate*.” (SW)

Academic researcher RV valued how the lived experience researchers helped her view the transcript through different lenses.

*“As I don’t have lived experience of prison or negative experiences with officers, I might take these interviews more at face value. It is important to note that the prison officers treated me as a full human being, we had informal chats before and after the interviews - for example one of the officers had a son living near me, which instantly created rapport.”*

The different reactions to, and interpretations of, the same interview show the benefit of including lived experience researchers in the analysis. Matching their lived experience with the accounts of prison officers offered a level of understanding (and scepticism) that would not have occurred if they had not also been involved in data analysis. It also points to the different ways prison officers present themselves to researchers from ‘outside’ and people ‘inside’ prison.

#### Ongoing learning

Involving lived experience researchers in this research project shows the ongoing learning that occurs both for academic researchers and lived experience researchers.

“*This project has made me question myself in many ways. When writing up the interview notes, I found myself reflecting on how going back into prison as a researcher makes you look at it from a different side. I saw first-hand, the lack of communication between officers, and realised that it was often a failing of the system, rather than a personal attack on the prisoners*.” (SW)

“*I went into this project thinking I had quite an open mind, but the more involved I've been and reflected on what others and myself are thinking, I have been able to understand my own biases more. Particularly towards prison officers. I understand this is down to my own treatment and that of others, and I often found myself feeling quite bitter and angry towards them and the criminal justice system*” (AB)

For the lived experience researchers involvement in prison research could also be therapeutic, as returning to prison as a researcher offered a way to heal some wounds. It highlighted some of their biases, particularly against prison officers, which is not surprising having experienced prison from the ‘other’ side.

The benefits of including lived experience researchers in research are also experienced by academic researchers, for example RV reports what she gained from the experience:

*“I personally feel this research was as much about the findings, as it was about the development of lived experience researchers, but it is not always easy to let go the reins. I conducted patient interviews both with lived experience researchers and on my own, and I can definitely see the value of having lived experience researchers in the room. They level the playing field and break down barriers between participants and researchers.” (RV)*

All three lived experience researchers emphasised how powerless they felt when they were in prison. They described feeling gaslighted, as there are limited ways to gather information or to find the ‘truth’. Their involvement in this research revealed to them that there are different ‘truths’ in prison, and the deeply ingrained ‘them’ vs. ‘us’ between prison officers and prisoners, is something that people with lived experience of prison carry with them for a long time. Involvement in research can alter this experience:

“*Through this research I learnt that there’s people like yourself [RV] trying to make situations better for prisoners, plus it’s possible for people like me to be invited back into prison as a researcher which I never thought possible, didn’t think it would have been possible a few years ago*” (AX)

This project was an opportunity for lived experience researchers to use their lived experience of prison to turn some of the negatives into a positive. Their past experiences in prison were acknowledged and considered meaningful in the design and conduct of this study. It also became clear that not only was the project about learning how cancer is managed in prison, an element of it evolved into developing lived experience researchers and learning from each other during the entire research process.

The process of having the four reflective discussions involved in writing this paper enhanced the input of the lived experience researchers and encouraged dialogue between them and the academic researchers. Written reflections encouraged lived experience researchers to think about what being involved in the study had meant to them individually. Our group discussions about those reflections strengthened the relationship between us as academic and lived experience researchers and highlighted how we can be in the same research environment, but experience and interpret the same situation differently. Thus, analysing the same data as a team encouraged a balanced interpretation of the interview data.

### Lessons learned

Our experience highlights how conducting healthcare research with lived experience researchers of a prison environment requires careful thought on how to include them in a meaningful and appropriate way. Below we outline lessons we learned from this project that may be useful for future researchers to consider when involving people with lived experience of prison in health-related research studies.

#### Developing open, honest and equal working relationships

Lived experience and academic researchers are experts in different things, and this needs to be acknowledged and discussed. Academic researchers need to be willing to share power, for example by letting lived experience researchers take the lead on interviews. Afterwards academic researchers need to take the time to debrief and offer constructive guidance to lived experience researchers to develop them as researchers. An issue pertinent to the involvement of lived experience researchers who have been in prison is an ingrained feeling that, because of their prison history, their opinions and perspectives somehow are less valid. As a research team we spent considerable time unpacking this issue. Respecting everyone on the team and developing working relationships where everyone feels on an equal footing is important. This is the case for lived experience researchers in general but, due to the stigma surrounding prison, specifically for researchers with lived experience of prison. It is important to both value the perspectives of the entire research team and also to ensure these perspectives are reflected in research outputs.

#### Skills development of academic and lived experience researchers

Including people with lived experience of prison in health-related research needs to be more than a tick box exercise if the benefits are to be fully realised. To unlock these requires careful planning, a commitment to shared decision-making and shared learning. Training and ongoing support for lived experience researchers is essential to achieving a good research outcome. Therefore, research training days and resources tailored to the specific project are needed and it is crucial to deliver these sessions at the right level and tone for the lived experience researchers involved. It might be difficult for them to identify what type of training they need or want, but ongoing dialogue between lived experience and academic researchers is again important to elucidate this. Guidance and support for academics on how to collaborate with lived experience researches is also helpful.

#### Commitment to building and maintaining relationships

Collaborative relationships between academic and lived experience researchers only work when there is a commitment to investing time and energy in the building of relationships. And this is something that takes time and cannot be rushed. Prison research requires much emotional labour, both from lived experience researchers as well as academic researchers. Therefore, it is important to be open about this, and to have safeguarding in place. Lived experience researchers received support from RDA and from academic researcher RV. Flexible funding schemes to build and maintain strong working relationships between lived experience and academic researchers would be extremely helpful, as initially the budget for our study did not include enough funds for this.

#### The importance of a third party as a go-between

Recruitment of people with lived experience of prison can be difficult [3]. In our study, the recruitment and support of lived experience researchers was conducted by Revolving Doors Agency. They fulfilled several functions we feel it is important to address when working with lived experience researchers. Firstly, due to their connections they were able to identify lived experience researchers with relevant experience to work with us. Secondly, they offered appropriate safeguarding for the lived experience researchers and served as their advocates. Lastly, they were a sounding board both for the lived experience and academic researchers, thereby playing an important role in the building and maintaining of relationships.

#### Putting “do no harm” into practice

An important ethical principle is to not do harm. While this is relevant to all research involving lived experience researchers, peer researchers with lived experience of prison are encouraged to share stories of their trauma and stigmatisation in prison. Lived experience researchers can feel used sharing their stories [6]. It is thus important to value these stories, but equally to make sure that lived experience researchers are not retraumatized through their involvement in research. Conducting qualitative research is emotive and has long lasting consequences beyond the conduct of interviews. Co-conducting interviews and analysing interview transcripts are, on the one hand, powerful ways for lived experience researchers to experience personal growth and develop research skills, but, on the other hand, unpleasant lived experience of the past can resurface because of this involvement. The writing of this paper demonstrates the importance of ongoing reflection and ‘checking in’ with lived experience researchers even after data collection has finished as being part of research can have powerful positive and negative consequences.

## Conclusion

With the right mindset from all parties involved (i.e. academic researchers, lived experience researchers and funders), and appropriate support, the involvement of people with lived experience of prison in research can encourage meaningful knowledge exchange and creation. Involving lived experience researchers in all stages provided valuable insight in the lived reality of prison. In the design stage this was reflected in the development of more pertinent interview questions and information sheets that were at the right level and tone for the intended audience. During the interview stage, lived experience researchers had the opportunity to practice their research skills but were also invaluable in their understanding of the research environment and some of the logistical challenges faced. The analysis sessions and continued discussions as a research team provided additional insight in the lived reality of prison. Interviews can be seen as snapshots of a research environment and comparing the experiences of both people working in prison and other prisoners to their own offered original and meaningful insight into the research topic. This involvement takes time and effort but, when done well, proves to be an enriching experience. Both academic and lived experience researchers learned from collaborating on this study and this collaboration significantly improved the relevance of our research. This collaborative approach is something that we as a research team encourage other researchers to take forward. Much research conducted in prison settings is critical of the criminal justice system. The involvement of people with lived experience of prison in research is one way to critically analyse and potentially bring about positive change within this system.

## Supplementary Information

**Additional file 1.**

## Data Availability

N.A.

## Abbreviations

EbE: Expert by experience
RDA: Revolving doors agency

## Footnotes

1 See: http://www.revolving-doors.org.uk/

2 For more information about the larger study see https://www.fundingawards.nihr.ac.uk/award/16/52/53.

3 For safeguarding reasons we deliberately do not reveal many details on the background of individual lived experience researchers.

4 This is a pseudonym.

5 During all the interview days with peer researchers, academic researcher RV reiterated that peer researchers SW and AX should let her know if they had had enough, if it was too intense or if they wanted to leave. Both peer researchers stated they were fine, but these types of check-ins are important. There are power dynamics within the research team as well. While this would not have negatively impacted the peer researcher’s participation in this project, these types of discussions need to be had with peer research members on an ongoing basis. As this is an ‘opportunity’ after prison, peer researchers might feel uncomfortable or ungrateful refusing to perform certain tasks and it is therefore essential to have safeguarding measures in place.

6 This comment refers to the fact that researcher RV is not a native English speaker and sometimes struggles understanding accents.

7 NB. Cowley et al. [12] use the term ‘service users’ as opposed to our term ‘lived experience researcher’

8 Face-to-face analysis sessions were planned but due to COVID-19 these had to move online. We found that these online analysis sessions were successful as we already established working relationships as a research team. Ideally, we would have had a mix of face-to-face and virtual analysis meetings.

9 This phrase was used repeatedly by an officer in their interview.
